# Hidden Metachronous Invasive Rectal Cancer after Repeated Endoscopic Treatment Following Ileorectal Anastomosis for Familial Adenomatous Polyposis: A Case Report

**DOI:** 10.70352/scrj.cr.26-0349

**Published:** 2026-09-17

**Authors:** Mamoru Sugihara, Kyota Tatsuta, Mayu Sakata, Toru Takagi, Yuya Iwase, Kosuke Sugiyama, Toshiya Akai, Yoshifumi Morita, Hirotoshi Kikuchi, Yoshihiro Hiramatsu, Hiroya Takeuchi

**Affiliations:** 1 Department of Surgery, Hamamatsu University School of Medicine, Hamamatsu, Shizuoka, Japan; 2 Department of Perioperative Functioning Care & Support, Hamamatsu University School of Medicine, Hamamatsu, Shizuoka, Japan

**Keywords:** familial adenomatous polyposis, ileorectal anastomosis, endoscopic surveillance

## Abstract

**INTRODUCTION:**

Patients with familial adenomatous polyposis (FAP) who undergo colectomy with ileorectal anastomosis (IRA) are at risk of adenoma recurrence and malignant transformation in the residual rectum. Advances in endoscopic techniques have enabled intensive surveillance with aggressive polypectomy to reduce the short-term risk of malignant progression. However, long-term management often requires a balance between endoscopic management and timely surgical intervention, and evidence to guide this balance remains limited.

**CASE PRESENTATION:**

A 54-year-old woman with FAP underwent total colectomy with IRA. During surveillance, an intramucosal rectal adenocarcinoma was detected at 65 years of age and resected transanally. Despite regular endoscopic treatment of recurrent lesions using endoscopic mucosal resection and argon plasma coagulation, the patient developed anal pain at 73 years of age. Imaging revealed a fluid collection suggestive of a posterior rectal wall abscess. Abdominoperineal resection was performed, and histopathological examination revealed invasive mucinous adenocarcinoma, leading to a diagnosis of metachronous rectal cancer. The patient has remained recurrence-free for 12 years postoperatively.

**CONCLUSIONS:**

This case highlights the limitations of long-term endoscopic therapy in patients with FAP following IRA. Repeated interventions may have contributed to chronic inflammation and mucosal alterations, which might have impaired visualization and complicated the detection of invasive carcinoma. Clinicians should avoid relying solely on endoscopic management and should regularly reassess the need for timely surgical intervention.

## Abbreviations

APC
: argon plasma coagulation
APR
: abdominoperineal resection
EMR
: endoscopic mucosal resection
FAP
: familial adenomatous polyposis
IPAA
: ileal pouch–anal anastomosis
IRA
: ileorectal anastomosis
LST-G
: granular-type laterally spreading tumor
NCCN
: National Comprehensive Cancer Network

## INTRODUCTION

FAP is an autosomal dominant hereditary disorder caused by pathogenic variants in the *adenomatous polyposis coli* gene and is characterized by the development of hundreds to thousands of adenomatous polyps throughout the colon. Without appropriate intervention, affected individuals face a near-inevitable lifetime risk of developing colorectal cancer.^1)^ Consequently, prophylactic colectomy is recommended to reduce this risk in patients with FAP.^2–4)^ Currently, total proctocolectomy with IPAA is the standard surgical approach for FAP.^2–4)^ Before the widespread adoption of IPAA, total colectomy with IRA was the most commonly performed procedure.^5)^ IRA remains a valid option in selected cases, particularly when postoperative bowel function or fertility preservation is prioritized.^6)^

Because the rectum is preserved after IRA, the risk of adenoma recurrence and malignant transformation in the remnant rectum persists, necessitating lifelong endoscopic surveillance after surgery.^7–9)^ Recent advances in endoscopic techniques have demonstrated that intensive surveillance combined with aggressive polypectomy can reduce the short-term risk of malignant progression in the remnant rectum.^10)^ However, the long-term consequences of repeated endoscopic interventions in the remnant rectum after IRA remain unclear. Herein, we report a case of metachronous invasive rectal cancer that was not detected during long-term, repeated endoscopic treatment after IRA for FAP.

## CASE PRESENTATION

At 53 years of age, the patient underwent colonoscopy following a positive fecal occult blood test, which revealed more than 100 colorectal adenomas, leading to a clinical diagnosis of FAP. Genetic testing for germline pathogenic variants in the *adenomatous polyposis coli* gene was not performed. At the time of diagnosis, no gastric adenomas, duodenal adenomas, or congenital hypertrophy of the retinal pigment epithelium were identified. At 54 years of age, she underwent prophylactic total colectomy with IRA, which was selected as an acceptable surgical option because the rectal polyp burden was limited (≤20 polyps) and was considered manageable by postoperative endoscopic surveillance. Histopathological examination of the resected specimen revealed 619 adenomatous polyps. The largest lesion was a 20 × 12-mm tubular adenoma with moderate-grade dysplasia located in the sigmoid colon. No malignant lesions were identified in the resected specimen. Only 1 small tubular adenoma with moderate-grade dysplasia was present in the remnant rectum; this lesion was removed by hot biopsy during surveillance colonoscopy 1 year after surgery.

After IRA, she underwent regular surveillance with colonoscopy and esophagogastroduodenoscopy, as well as periodic CT examinations to evaluate for intra-abdominal lesions. No intra-abdominal desmoid tumors or apparent neoplastic lesions in the remnant rectum were detected on CT during the follow-up period. Postoperative surveillance with esophagogastroduodenoscopy revealed no gastric or duodenal adenomas. Four years after the initial surgery, carcinoma of the ampulla of Vater was diagnosed, and pylorus-preserving pancreaticoduodenectomy was performed.

At 65 years of age, an LST-G involving more than half of the rectal circumference was detected and diagnosed as an adenocarcinoma on biopsy. Although proctectomy was recommended, the patient refused surgery due to substantial physical decline experienced after the initial procedure. Instead, transanal tumor excision was performed. The lesion was removed in a piecemeal manner, and macroscopically complete excision was considered to have been achieved. Histopathological examination revealed a well-differentiated adenocarcinoma arising in a tubular adenoma and confined to the mucosa. The vertical margin was negative; however, because the specimen was fragmented, the horizontal margin and the presence of residual adenomatous tissue at the margin could not be reliably assessed.

**Fig. 1:**
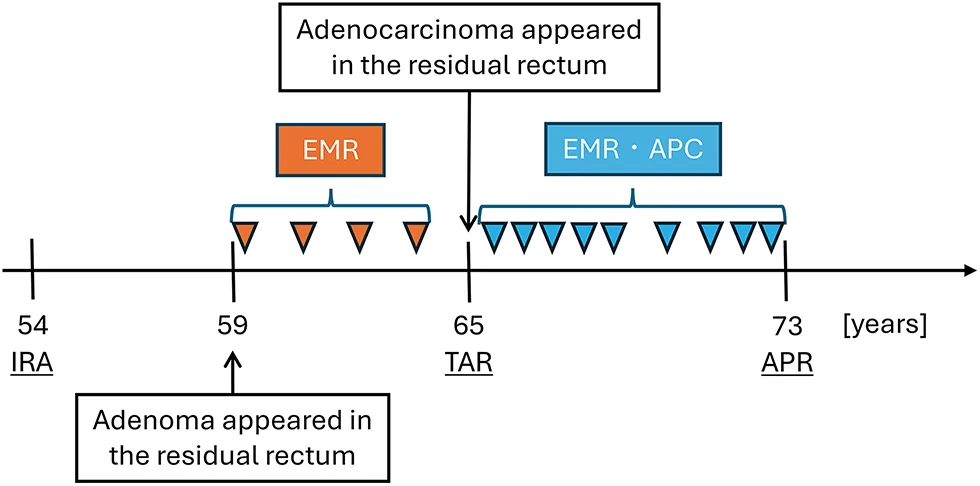
Clinical course during endoscopic surveillance after prophylactic colectomy. The patient underwent colectomy with IRA at 54 years of age. At 59 years of age, adenomas were detected in the residual rectum, and EMR was initiated. At 65 years of age, intramucosal rectal adenocarcinoma was diagnosed and treated with TAR. Thereafter, recurrent lesions were treated with EMR and APC during surveillance performed every 6 months. However, endoscopic control gradually became difficult, and APR was ultimately performed at the age of 73. APC, argon plasma coagulation; APR, abdominoperineal resection; EMR, endoscopic mucosal resection; IRA, ileorectal anastomosis; TAR, transanal resection

The patient subsequently continued endoscopic surveillance at 6-month intervals. Neither narrow-band imaging nor chromoendoscopy was used during surveillance; therefore, endoscopic assessment was based on conventional white-light imaging. During the entire surveillance period, 4 endoscopic sessions involving EMR alone were performed before transanal tumor excision, followed by 9 sessions involving both EMR and APC (**Fig. 1**). The lesions resected by EMR after transanal tumor excision were diagnosed as tubular adenomas with moderate- or high-grade dysplasia or as intramucosal adenocarcinoma. Because some lesions were resected in a piecemeal manner, the resection margins were frequently recorded as not assessable. APC was applied both around the EMR sites and directly to small lesions. No residual lesions requiring additional treatment were apparent endoscopically at these examinations.

**Fig. 2:**
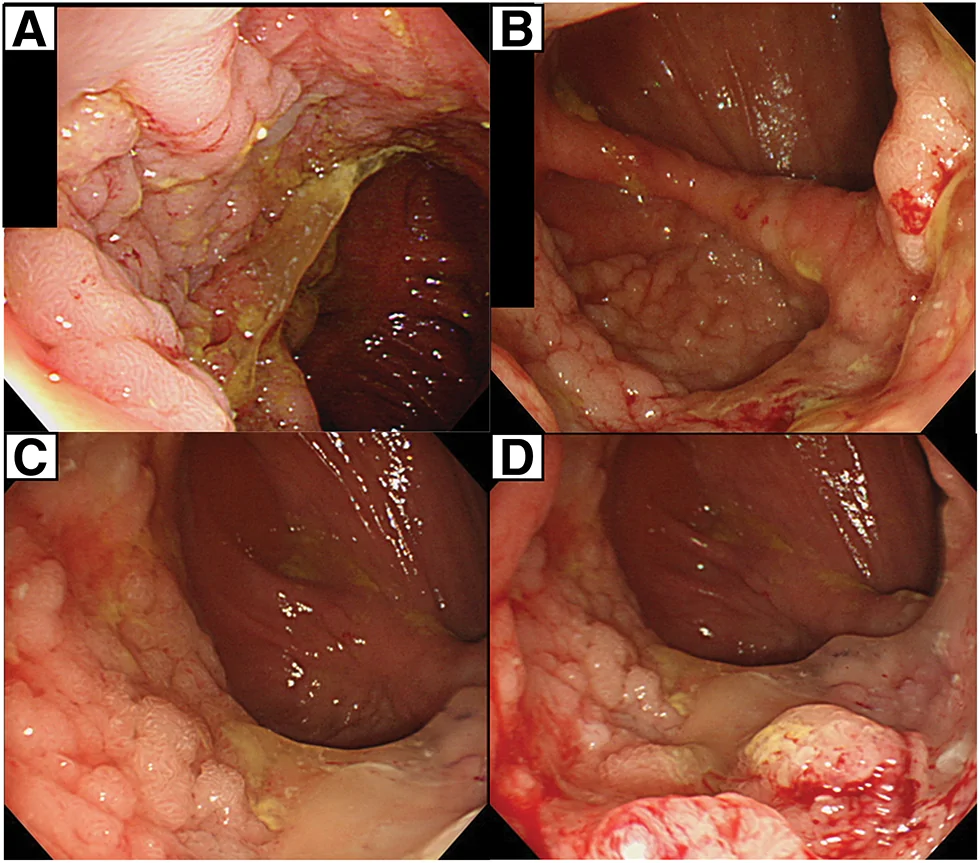
Endoscopic findings during surveillance. (**A**) Colonoscopy at the age of 65 revealed an LST-G occupying approximately half of the rectal circumference. (**B**) At the age of 69, multiple broad-based polyps were identified and treated with EMR and APC. (**C**, **D**) At the age of 73, follow-up colonoscopy demonstrated recurrence of the LST-G and additional broad-based polyps. APC, argon plasma coagulation; EMR, endoscopic mucosal resection; LST-G, granular-type laterally spreading tumor

**Fig. 3:**
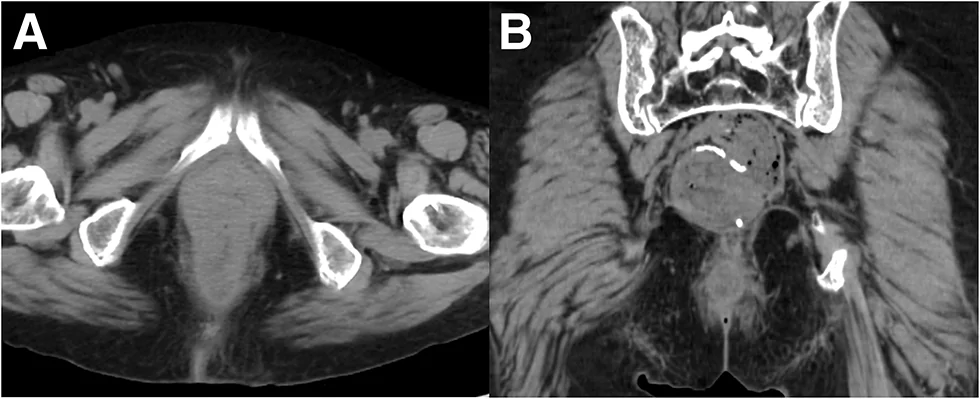
Pelvic CT imaging at the age of 73. (**A**) Axial view. (**B**) Coronal view. A 35 × 20-mm area with fluid collection was observed posterior to the rectal wall, accompanied by increased attenuation of the surrounding fat tissue, suggestive of a perirectal abscess.

**Fig. 4:**
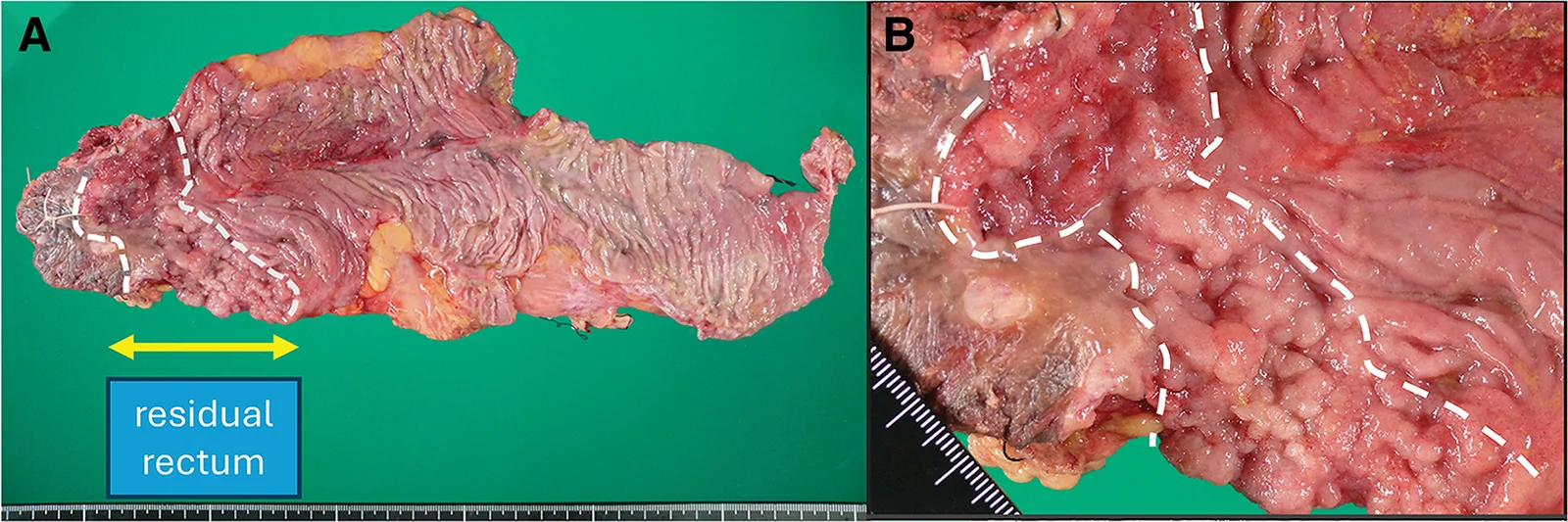
Resected specimen of the residual rectum. (**A**) Approximately 6 cm of the residual rectum is shown between the white dashed lines. An LST-G was observed, involving about half of the rectal circumference on the anal side and nearly the entire circumference on the oral side. (**B**) A fistula-like tract was observed extending from the distal margin of the LST-G lesion to the perianal skin. LST-G, granular-type laterally spreading tumor

At 73 years of age, follow-up endoscopy revealed an LST-G and a broad-based polyp, both of which were managed endoscopically (**Fig. 2**). Five months later, the patient presented with anal pain. Pelvic CT revealed a 35 × 20-mm fluid collection posterior to the rectal wall with surrounding fat stranding (**Fig. 3**). Pelvic MRI showed high signal intensity in the corresponding area on both T2-weighted and diffusion-weighted images. No definite invasive component had been recognized during the 6-month interval of endoscopic surveillance, and given the history of repeated endoscopic treatment, the lesion was initially suspected to represent an abscess or inflammatory change related to the previous interventions. As further endoscopic therapy was deemed unfeasible, definitive surgery was recommended. The patient subsequently underwent APR with an end ileostomy (**Fig. 4**).

**Fig. 5:**
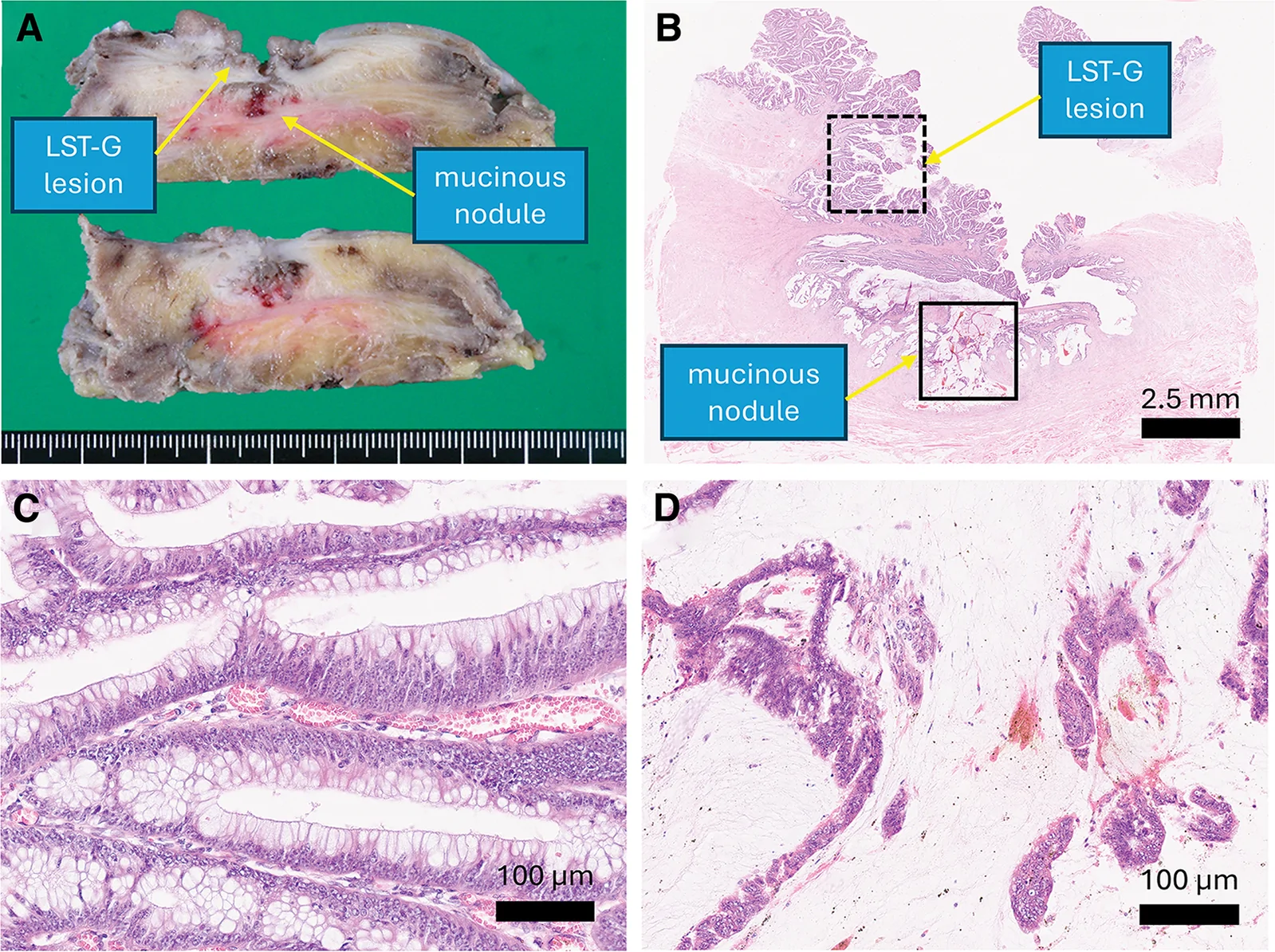
Histopathological findings in the residual rectum. (**A**) A mucinous nodule measuring approximately 15 × 8 mm was identified beneath the distal portion of the LST-G lesion, extending from the submucosa to the muscularis propria. (**B**) Histologically, the superficial layer contained well-differentiated adenocarcinoma and adenomas with moderate- to high-grade dysplasia (black dotted box), whereas the deeper layer showed invasive mucinous adenocarcinoma (black solid box). No clear histological continuity was identified between the superficial and deeper components. (**C**) Magnified view of the black dotted box in panel (**B**), showing well-differentiated adenocarcinoma and adenomatous glands with moderate- to high-grade dysplasia in the superficial layer. (**D**) Magnified view of the black solid box in panel (**B**), showing invasive mucinous adenocarcinoma characterized by irregular malignant glands and clusters of atypical epithelial cells within abundant extracellular mucin in the deeper layer. LST-G, granular-type laterally spreading tumor

Histopathological examination of the resected specimen revealed widespread well-differentiated adenocarcinoma and tubular adenomas with moderate- to high-grade dysplasia throughout the remnant rectum. Additionally, an invasive mucinous adenocarcinoma was identified in the deeper layer of the LST-G, infiltrating the muscularis propria and forming a mucinous nodule associated with a fistulous tract opening in the perianal skin (**Fig. 5**). The lesion initially suspected to be an abscess on CT was ultimately diagnosed as invasive rectal adenocarcinoma with mucinous features. The proximal and distal margins were negative, and no carcinoma was exposed at the radial surgical margin. The resection margin of the fistulous tract, including the perianal skin margin, was also negative; therefore, an R0 resection was achieved. No lymphovascular invasion or lymph node metastasis was observed. Molecular testing of the tumor, including microsatellite instability testing, was not performed. The postoperative course was uneventful, and the patient has remained recurrence-free for 12 years since the last surgery.

## DISCUSSION

This case describes a patient with FAP who underwent IRA and was managed with long-term endoscopic surveillance and treatment of residual rectal lesions for nearly 2 decades. During the follow-up, a rectal lesion with fluid collection was initially suspected to represent an abscess or inflammatory change possibly associated with repeated endoscopic interventions. However, histopathological examination revealed a rectal adenocarcinoma infiltrating the lower border of the muscularis propria. This case highlights the insidious occurrence of invasive malignancy despite the absence of clear endoscopic evidence after IRA, emphasizing the need for timely surgical intervention when managing residual rectal lesions.

The reported cumulative risk of rectal cancer after IRA ranges from 6% to 33% across heterogeneous observational studies.^3)^ In a retrospective cohort of 140 patients, the cumulative risk of rectal cancer reached 24% and the cumulative risk of secondary proctectomy reached 53% at 30 years after IRA.^7)^ Nevertheless, repeated endoscopic treatment has shown favorable long-term outcomes in selected patients.^10–12)^ Intensive endoscopic management has enabled many patients to avoid or postpone completion proctectomy, although secondary rectal cancer still occurred in 6.1% of patients after a median follow-up of 17.1 years.^11)^ Furthermore, only 1 of 46 patients with IRA required completion proctectomy during 5 years of intensive endoscopic treatment in a prospective multicenter study.^10)^ These findings suggest that repeated endoscopic treatment is an effective strategy for selected patients, although its lifelong oncological safety remains uncertain.

In the present case, regular endoscopic interventions were performed every 6 months to control recurrent lesions. Nevertheless, invasive rectal cancer extending into the deep muscularis propria eventually developed. Repeated endoscopic treatment may have contributed to local inflammation, fibrosis, and regenerative changes, which might have impaired mucosal visibility and obscured deeper tumor invasion. Similar diagnostic challenges have been described in inflammatory bowel disease,^13)^ and comparable limitations may arise during long-term endoscopic management after IRA in patients with FAP. Furthermore, because the remnant rectal mucosa harbors diffuse oncogenic potential and often contains numerous adenomas, accurately assessing the malignant potential and depth of invasion of individual lesions by endoscopy alone remains challenging.^14)^ Histologically, the superficial mucosa contained well-differentiated adenocarcinoma and a high-grade adenoma, whereas mucinous adenocarcinoma was identified only in the deeper layers without clear continuity. One possible explanation is that an occult mucinous component had already been present beneath a previously treated lesion and subsequently progressed within the deeper bowel wall after EMR or APC of the superficial lesion. Although no mucinous component was identified in specimens obtained by transanal excision or subsequent EMR, APC destroys tissue without histological evaluation, which raises the possibility that a pre-existing mucinous component beneath an APC-treated lesion escaped pathological detection.

Previous reports have demonstrated that regular endoscopic surveillance after IRA facilitates the early detection of rectal cancer and may help patients avoid repeat bowel resection, whereas interruption of surveillance increases the risk of invasive carcinoma.^11,15,16)^ In contrast, the present case raises the possibility that repeated endoscopic treatment might occasionally hinder endoscopic assessment and obscure deeply invasive carcinoma. To our knowledge, few reports have highlighted this potential pitfall of long-term endoscopic surveillance after IRA in patients with FAP. Accordingly, these findings suggest that clinicians should recognize the limitations of repeated endoscopic treatment and consider timely completion proctectomy when surgical indications are met. Current European and NCCN guidelines recommend considering completion proctectomy when rectal polyposis is no longer manageable endoscopically or when high-risk adenomas are identified.^3,4)^ In the present case, completion proctectomy was recommended after intramucosal carcinoma was diagnosed but was declined because of the patient’s previous postoperative experience. The apparent control of the rectal lesions with repeated endoscopic treatment may have influenced the decision to continue surveillance rather than proceed immediately to completion proctectomy. Given that fear of reoperation is common among patients with FAP,^17,18)^ careful decision-making is essential to avoid delaying appropriate surgical intervention.

To support this decision-making, involvement of a multidisciplinary team may be beneficial. In the present case, the treatment strategy for intramucosal rectal adenocarcinoma diagnosed at 65 years of age was discussed primarily between the colorectal surgical team and the patient; however, no formal multidisciplinary conference involving endoscopists or specialists in hereditary colorectal disease was held. Previous studies have shown that multidisciplinary management improves the quality of care for patients with hereditary colorectal cancer syndromes.^19)^ As treatment strategies continue to evolve with advances in endoscopic therapy, minimally invasive surgery, and perioperative care, decisions regarding the timing and type of surgery should incorporate the colorectal phenotype, genotype–phenotype correlations, and patient-related factors.^3)^ Accordingly, multidisciplinary evaluation involving gastroenterologists, endoscopists, colorectal surgeons, and specialists in hereditary colorectal disease should be considered, as it may facilitate individualized decision-making and help optimize long-term management after IRA.

## CONCLUSIONS

Determining the optimal timing to transition from endoscopic management to surgical treatment remains a critical challenge in the long-term care of patients with FAP after IRA. When repeated endoscopic interventions are required over an extended period, clinicians should recognize the limitations of endoscopic management and reassess the need for timely surgical resection of the remnant rectum.

## DECLARATIONS

### Availability of data and materials

Not applicable, as this is a case report and all relevant data are included in this article.
